# How Disgust and Fear Influence Strategic Aspects of Arithmetic Performance in Children and Adolescents

**DOI:** 10.3390/jintelligence14100255

**Published:** 2026-10-11

**Authors:** Camille Lallement, Patrick Lemaire

**Affiliations:** Centre de Recherche en Psychologie et Neurosciences, Aix-Marseille Université & CNRS UMR 7077, 13001 Marseille, France

**Keywords:** emotion, cognition, arithmetic, problem-solving, strategies, children and adolescents

## Abstract

This study is the first to combine a strategy perspective with a typological approach to emotions to determine how disgust and fear influence arithmetic performance in children and adolescents. In Experiment 1, 10–11-year-old (*N* = 32) and 14–15-year-old (*N* = 32) participants were asked to choose between two rounding strategies, whereas in Experiment 2, a different sample of 10–11-year-old (*N* = 32) and 14–15-year-old (*N* = 32) participants executed instructed rounding strategies to estimate the sums of two-digit addition problems following either emotionally neutral or negative (disgust- or fear-inducing) pictures. Negative emotions led participants to select the better strategy less frequently and different patterns of associations between problem characteristics and better-strategy selection were observed across emotion conditions: whereas selection was associated with the difference of deviations, a diagnostic strategy-selection cue, in the neutral condition, it was associated with problem-magnitude information in the negative-emotion condition. Negative emotions also slowed strategy execution, with participants being slower under disgust than under fear. Moreover, disgust reduced response accuracy relative to the neutral condition in 10–11-year-old children but not in 14–15-year-old adolescents. In contrast, the effects of negative emotions on better-strategy selection and strategy execution latencies did not significantly differ between age groups. These findings have important implications for furthering our understanding of how negative emotions influence arithmetic performance in particular, and cognitive performance in general, both in children and adolescents.

## 1. Introduction

Emotions are known to influence cognitive performance in children and adolescents, although this influence changes with age and different task parameters such as task difficulty (see Bell, 2024; Bell et al., 2019, for reviews). Effects of emotions on children’s cognitive performance have recently been found to be mediated by the strategies that children use to accomplish a cognitive task, such that for example negative emotions lead children to select the better strategy less often on each item and to execute each strategy more poorly (Lemaire, 2026). Unknown however is how negative emotions influence strategy selection and strategy execution in children. For example, we ignore if negative emotions lead children to more poorly analyze the problem characteristics to calibrate their strategy choices to problem features. Also, although some findings suggest that different negative emotions have different effects on arithmetic performance (Lallement & Lemaire, 2025), we do not know whether different negative emotions change how children execute strategies when all other strategic aspects of cognitive performance are controlled. To address these questions, we examined arithmetic performance by integrating a strategy-based framework with a typological perspective on emotions. We begin by considering evidence concerning the influence of emotions on cognitive processes more broadly, before reviewing research specifically examining how negative emotions affect arithmetic performance in children.

### 1.1. Effects of Emotions on Cognition

A substantial body of research has examined the interplay between emotion and cognition in adults across a variety of cognitive domains (see De Houwer & Hermans, 2010; Lemaire, 2021; Robinson et al., 2013, for reviews). This research indicates that emotional states and stimuli can either benefit or disrupt cognitive functioning depending on the circumstances. Evidence from memory research illustrates this dual influence. Emotional material, including words, sentences, and pictures, is generally remembered more successfully than neutral material (e.g., Chapman et al., 2013; Davidson et al., 2006; Davis et al., 2019; Kensinger & Corkin, 2004; Le Bigot et al., 2018; Talamini et al., 2022; Yeung & Fernandes, 2021), whereas negative emotional states such as stress can reduce memory performance (e.g., Kuhlmann et al., 2005; Payne et al., 2006). Attentional processes provide one prominent account of these contrasting effects (Okon-Singer et al., 2015; Pessoa, 2009; Verbruggen & De Houwer, 2007). Because emotional information tends to attract attention automatically, it can facilitate performance when it is relevant to current task goals. When emotional information is unrelated to the task, however, this attentional capture can divert resources from task processing and consequently hinder performance.

In children, emotions have also been shown to influence cognitive performance in a variety of domains, including attention, memory, reasoning, or problem-solving (see Bell, 2024; Bell et al., 2019, for reviews). Moreover, the negativity bias (i.e., being more attracted to and influenced by negative emotions than positive or neutral emotions) robustly reported in young adults has likewise been demonstrated in children from an early age (see Vaish et al., 2008, for a review). For example, in the domain of attention, 7–10-year-old children showed enhanced attentional processes (as seen in larger amplitudes of attention-related ERPs such as P2 and early and late P3a) in response to emotionally negative sounds compared to neutral sounds, while instructed to focus on a silent video clip and to ignore the concurrently presented auditory stimuli (Bonmassar et al., 2020). Emotions have also been shown to influence memory early in development (see Hamann & Stevens, 2013, for a review). Indeed, from as early as 5–6 years of age, children remembered emotionally negative stories better compared to emotionally positive or neutral stories (Van Bergen et al., 2015), recalled emotional words more accurately than neutral words between the ages of 8 and 11 (Massol et al., 2020), and continue to show this emotional enhancement of memory during adolescence (Krauel et al., 2007). Finally, emotions also influence problem-solving performance in children (see Hannula, 2015, for a review). For example, 6–7-year-old children performed worse on problems with cubes, involving spatial manipulation and analysis, after listening to an emotionally negative story than after an emotionally positive story (Rader & Hughes, 2005).

As in adults, effects of emotions in children are generally explained by attentional mechanisms, whereby emotionally salient information captures children’s attentional resources and modulates cognitive processing. Importantly, links between emotion and cognition in childhood have been particularly well documented in educational and learning contexts, highlighting their critical role in academic development (see Goetz & Hall, 2013; Pekrun & Linnenbrink-Garcia, 2012, 2014, for reviews). This makes the study of emotion–cognition interactions especially relevant in school-related domains, such as mathematics, and arithmetic more specifically, which constitutes a core academic skill and remains essential for adults’ everyday functioning.

### 1.2. Effects of Emotions on Children’s Arithmetic Performance

#### 1.2.1. Effects of Emotional Traits

One line of research on the relations between emotions and arithmetic during childhood has focused on children’s emotional traits, particularly achievement emotions—emotions related to achievement activities and their success and failure outcomes (Pekrun et al., 2017). These emotions have been extensively studied in relation to arithmetic learning (see Batchelor et al., 2019, for a review). Studies have shown that positive achievement emotions, such as enjoyment and pride, reliably predict higher subsequent mathematics achievement, whereas negative emotions such as anger, shame, or boredom, predict poorer later performance in both children and undergraduates (e.g., Pekrun et al., 2017, 2023a, 2023b; Villavicencio & Bernardo, 2016). Longitudinal evidence further indicated that these relations were reciprocal: Higher achievement predicts positive emotions, which in turn promote achievement, whereas negative emotions both predict and are reinforced by lower performance (e.g., Lichtenfeld et al., 2023; Pekrun et al., 2023b). Importantly, these reciprocal links are mediated by children’s perceived competence and self-appraisals of mathematical ability (e.g., Forsblom et al., 2022; Van der Beek et al., 2017).

Within this literature on emotion–arithmetic relations during childhood, mathematics anxiety—feelings of tension and apprehension specific to mathematics—has received the largest empirical attention, as a key emotional trait shaping arithmetic learning and performance (see Cipora et al., 2022; Dowker et al., 2016; Luttenberger et al., 2018; Mammarella et al., 2019, for reviews). Across grade levels, studies consistently reported a robust negative association between math anxiety and math achievement, such that highly math-anxious children perform more poorly than their low-anxious peers (e.g., Passolunghi et al., 2019; Pellizzoni et al., 2022; Živković et al., 2023; see Barroso et al., 2021; Zhang et al., 2019, for meta-analyses). This decrease in performance is commonly attributed to intrusive, task-irrelevant thoughts that capture attentional and working memory resources required for effective problem solving, thereby reducing the resources available for mathematical processing. Although this body of work provides compelling evidence that emotions influence children’s arithmetic performance, it has primarily relied on individual differences in trait-like emotional dispositions. Complementary evidence comes from experimental studies that manipulate children’s emotional states through emotion induction procedures.

#### 1.2.2. Effects of Emotional States

Several experimental studies have shown that induced emotions influence mathematical cognition in adults. Most have demonstrated deleterious effects of irrelevant positive and negative emotions on performance in various arithmetic tasks (e.g., Framorando & Gendolla, 2018, 2019; Kleinsorge, 2007, 2009; Lallement & Lemaire, 2021, 2025; Lemaire, 2024; Liu et al., 2021; Schimmack & Derryberry, 2005; Zhu et al., 2021, 2022, 2024).

Fewer experimental studies have been carried out in children and have sometimes led to conflicting results (Kucian et al., 2018; Lemaire, 2022, 2026; Rubinsten & Tannock, 2010; Viesel-Nordmeyer & Lemaire, 2025). Using the same paradigm (i.e., children were asked to solve arithmetic problems following display of emotional or neutral words), Rubinsten and Tannock (2010) and Kucian et al. (2018) reported contradictory results. Rubinsten and Tannock found that 7–13-year-old children were slower on solving arithmetic problems following emotionally negative words than after neutral words whereas Kucian et al. did not find emotional priming effects on arithmetic performance. Lemaire (2022) asked children and adolescents aged 8 to 15 to verify simple addition problems (e.g., 3 + 4 = 7, True or False?) that were presented superimposed on emotionally neutral or negative pictures. Participants in all age groups performed worse under negative than under neutral conditions. Interestingly, these deleterious effects of negative emotions decreased as children grow older (see Viesel-Nordmeyer & Lemaire, 2025, for similar findings). While these studies provide valuable evidence that emotions can influence children’s and adolescents’ arithmetic performance, they also motivate a closer examination of the mechanisms responsible for these effects.

### 1.3. How Do Emotions Affect Children’s Arithmetic Performance?

The detrimental influence of task-irrelevant emotions on arithmetic performance has generally been interpreted in terms of attentional capture, in both adults and children. According to this account, negative emotional information draws attentional resources away from the arithmetic task, leaving fewer resources available for task processing. More recently, researchers have examined arithmetic strategies to identify more precisely which components of performance are affected by this competition for cognitive resources (e.g., Lallement & Lemaire, 2021; Lallement & Lemaire, 2025; Lemaire, 2024). Findings in adults indicate that negative emotions can affect multiple components of strategic performance: individuals use a narrower range of strategies, favor easier strategies, show poorer strategy execution, and choose the better strategy for individual problems less frequently (Lemaire, 2024). Considerably less is known about these strategic effects during childhood. One recent study addressed this question by asking 8–14-year-olds to estimate the sums of two-digit addition problems (e.g., 32 + 56) in neutral and negative emotional conditions (Lemaire, 2026). Negative emotions were associated with less frequent selection of the better strategy and less efficient execution of the available strategies. In contrast to adults, negative emotions have no influence on children’s strategy repertoire or distribution. Interestingly, deleterious effects of negative emotions on strategic aspects of arithmetic performance decreased as children grow older.

Previous findings did not enable us to know how children and adolescents were led to select and execute strategies more poorly while solving arithmetic problems under negative emotions. Also, they did not enable us to know whether different emotions have different effects on children’s strategy selection and execution. For example, we do not know whether negative emotions impair strategy selection because children become less consistent in using the same relevant problem characteristic across problems. Or, do they change the information on which they base their decisions, relying on different—and potentially less diagnostic—problem characteristics to select a strategy? One aim of the present study is to address these issues.

A further limitation of previous research is that it remains unclear whether distinct negative emotions differentially affect strategic aspects of arithmetic performance. This is the result of previous studies adopting a dimensional approach to emotions, characterizing emotions along continuous dimensions such as valence (i.e., the positive or negative quality of an emotion) and arousal (i.e., its intensity). This approach enabled to address important issues such as whether emotions influence arithmetic performance and whether these effects vary with age. However, a typological approach to emotions which conceptualizes emotions as distinct and discrete categories is necessary to gain a more precise understanding of how specific emotions influence children’s arithmetic performance. According to Pekrun’s Control-Value Theory (CVT; Pekrun, 2006, 2019; Pekrun & Linnenbrink-Garcia, 2012), discrete negative emotions can differ in their effects on learning and performance because they differ in their motivational and cognitive consequences. Although fear and disgust examined in the present study were induced by task-irrelevant pictures and therefore are not achievement emotions as defined within the CVT, this framework provides a relevant theoretical framework for considering the possibility that discrete negative emotions may differentially affect cognitive performance.

Importantly, developmental research suggests that children’s responses to negative emotions are not necessarily uniform across discrete emotions. Children progressively develop the ability to recognize and differentiate emotions such as fear, disgust, anger, and sadness. Emotion recognition continues to improve throughout childhood, although recognition accuracy varies depending on the type of emotion and children’s age (e.g., Cuciniello et al., 2025; Riddell et al., 2024). Moreover, fear and disgust may have distinct effects on children’s attention and emotional responses, suggesting that their cognitive consequences cannot be fully reduced to their shared negative valence (e.g., Reynolds & Askew, 2019). More broadly, developmental research has shown that emotion regulation and executive functions are closely related throughout childhood and adolescence, and that these relations may vary depending on the type of emotions (e.g., Fombouchet et al., 2024; Halse et al., 2024). Thus, previous developmental works provide reasons to expect emotion-specific effects on cognitive processes in children. Whether such differences extend to the strategic processes involved in arithmetic performance, however, remains unknown.

Evidence from adults suggests that the strategic consequences of negative emotions may vary with specific emotions (Lallement & Lemaire, 2025). In particular, disgust was associated with less frequent selection of the better strategy and slower execution of instructed strategies. Fear, in contrast, did not significantly affect strategy selection but produced greater slowing during strategy execution than disgust. Sadness had no influence on either strategy selection or execution. However, no studies examined how different emotions affect strategic aspects of children’s performance while children are solving arithmetic problems. Thus, we do not know if different negative emotions, such as disgust and fear, have the same or different effects on strategic aspects of children’s arithmetic performance, and whether this changes with children’s age. To fill this gap, the present study examined arithmetic strategies while distinguishing between discrete negative emotions.

### 1.4. Overview of the Present Study

This study pursued three main objectives: (a) to identify the mechanisms underlying the detrimental effects of negative emotions on better-strategy selection, (b) to examine whether strategic arithmetic performance is affected differently by distinct negative emotions, and (c) to investigate whether these emotion effects vary with age. Based on previous research on emotion and arithmetic (e.g., Kucian et al., 2018; Lemaire, 2022, 2024, 2026; Rubinsten & Tannock, 2010), emotions were induced on each trial while participants performed the arithmetic task, an approach previously shown to be sensitive to emotion-related changes in children’s arithmetic performance. Participants aged 10–11 and 14–15 years either selected the better of two rounding strategies (Experiment 1) or applied an instructed rounding strategy (Experiment 2) to estimate the sums of two-digit addition problems (e.g., 21 + 47). In both experiments, each arithmetic problem was preceded by a picture that was either emotionally neutral or designed to elicit disgust or fear.

This study used the same methodological approach as Lallement and Lemaire (2025) and extended this previous study in several important ways. Whereas Lallement and Lemaire’s study documented effects of discrete negative emotions on young and older adults’ arithmetic performance, the present study examined whether these emotion-specific effects are already present during late childhood and adolescence, a developmental period characterized by ongoing changes in strategy use, arithmetic proficiency, attentional control, and emotion regulation. Moreover, the present study went beyond documenting performance differences by investigating the problem characteristics that children and adolescents use to select strategies, thereby identifying the mechanisms through which negative emotions alter strategy selection. Finally, by comparing children and adolescents, the present study tested whether the strategic effects of fear and disgust change across development, providing new evidence on how emotion–cognition interactions evolve with age during childhood.

Thus, beyond replicating the detrimental effects of induced negative emotions on children’s arithmetic performance (Lemaire, 2022, 2026; Rubinsten & Tannock, 2010; Viesel-Nordmeyer & Lemaire, 2025), the present study tested three sets of original hypotheses. Our first hypothesis, based on the attentional capture account and previous evidence of emotions influencing strategic aspects of children’s arithmetic performance, concerned the mechanisms by which negative emotions disrupt children’s ability to select the better strategy to solve arithmetic problems. We hypothesized that negative emotions, by capturing attentional resources, would lead participants to rely less on relevant problem characteristics and instead to rely on more salient but less informative problem features, such as problem size (i.e., the magnitude of the operands and, consequently, of the sum), to select strategy on each problem. Such a hypothesis would be supported by a change in the best predictor of better strategy selection under negative emotions compared to a neutral emotion condition.

Our second hypothesis drew on the CVT and prior evidence from adults to predict how distinct negative emotions would affect strategic arithmetic performance in children. Because fear and disgust differ in their cognitive and motivational characteristics (Pekrun, 2006, 2019; Pekrun & Linnenbrink-Garcia, 2012), and because recent findings have shown that these emotions exert distinct effects on adults’ strategy selection and strategy execution (Lallement & Lemaire, 2025), we hypothesized that they would also differentially affect children’s strategic aspects of performance. Although the adult findings suggest that fear and disgust may impair different strategic components, it remains unclear whether the same pattern would emerge in children.

Our third hypothesis was based on previous developmental findings showing that the deleterious effects of negative emotions on children’s arithmetic performance decrease with age (e.g., Lemaire, 2022; Viesel-Nordmeyer & Lemaire, 2025). Because the present study aimed to identify the strategic mechanisms underlying these effects, we hypothesized that negative emotions would disrupt both strategy selection and strategy execution less in adolescents than in younger children. In other words, age-related reduction in the impact of negative emotions on arithmetic performance should be accompanied by a weaker influence of emotions on the strategic processes supporting performance. Furthermore, because discrete emotions differ in their cognitive and motivational characteristics (Pekrun, 2006, 2019; Pekrun & Linnenbrink-Garcia, 2012; Lallement & Lemaire, 2025), fear and disgust may exhibit different developmental patterns in their effects on strategy selection and execution. However, given the lack of developmental evidence comparing these two emotions, no specific prediction was made regarding the direction of these potential differences.

## 2. Experiment 1: Effects of Negative Emotions on Strategy Selection in Children and Adolescents

### 2.1. Method

#### 2.1.1. Participants

A total of 128 participants were recruited, with 64 assigned to Experiment 1 and 64 to Experiment 2. Participants attended public schools located in the South of France; their characteristics are reported in Table 1. Sample size was established a priori using G*Power 3 (Faul et al., 2007). Previous research examining emotion effects on arithmetic performance in children reported effect sizes ranging from *η*^2^*p* = 0.351 to 0.710 (Lemaire, 2022, 2026). Based on the smallest of these effect sizes (*η*^2^*p* = 0.351), a design including age as a between-participant factor and emotion and strategy as within-participant factors required 40 participants (20 per age group) to achieve 96% statistical power. We increased the sample to 64 participants per experiment, with 32 participants in each age group^1^. Parents provided written informed consent, and participants provided oral assent. The two experiments received approval from the French National Ethics Committee (Ref #: CNRIPH 2022-04-14-014).

#### 2.1.2. Stimuli

*Arithmetic problems*. The task comprised 64 two-digit addition problems displayed in the conventional *a + b* format (e.g., 21 + 47). Following established methodological practices in arithmetic research (see Cohen Kadosh & Dowker, 2015; Gilmore et al., 2018; Knops, 2019, for overviews), problem construction followed several constraints. Neither operand ended in 0 or 5, the two operands did not contain identical digits in corresponding decade or unit positions (e.g., 21 + 27), no operand contained repeated digits (e.g., 22 + 47), and problems with identical operands (e.g., 21 + 21) were excluded.

For half of the problems, the first operand had a unit digit below 5 and the second a unit digit above 5 (e.g., 21 + 47); this arrangement was reversed for the remaining problems (e.g., 57 + 31). Although the most accurate estimate for such problems can be obtained by rounding one operand down and the other up to the nearest decade, participants were instructed not to use this mixed-rounding strategy. This restriction was introduced to increase the demands associated with strategy selection. Indeed, previous research has shown that allowing mixed rounding makes selection highly straightforward, with participants choosing the better strategy on more than 95% of problems (e.g., Lemaire et al., 2004). Participants were asked to provide the better estimate on each problem using either a rounding-down (i.e., RD; rounding both operands down to their nearest decades) or a rounding-up strategy (i.e., RU; rounding both operands up to their nearest decades). The rounding-down strategy produced the estimate closest to the exact sum for half of the problems (e.g., estimating 21 + 47 as 20 + 40), whereas rounding up both operands was more accurate for the remaining problems (e.g., estimating 34 + 29 as 40 + 30). The 64 problems were divided into two balanced sets of 32 trials. Within each set, half the problems were paired with neutral pictures (e.g., a car), whereas the remaining problems were paired with negative pictures intended to elicit either fear (e.g., a snake) or disgust (e.g., clogged toilets). Across the resulting problem categories (Rounding-Down/Rounding-Up × Neutral/Negative), stimuli were equated with respect to (a) the position of the larger operand, which appeared equally often on the left and right, (b) the magnitude of the exact sums, and (c) the mean percentage deviation of the estimates from the exact sums, computed as [(estimate − exact sum)/exact sum] × 100.

*Pictures*. Sixty-four pictures were selected from the Nencki Affective Picture System (NAPS, Marchewka et al., 2014). In Nencki Affective Picture System, valence ranges from 1 (very negative) to 9 (very positive), and arousal ranges from 1 (relaxed) to 9 (aroused). The picture set comprised an equal number of neutral (*mean* valence = 3.7, *SD* = 0.7; *mean* arousal = 2.5, *SD* = 0.6) and negative stimuli (mean valence = 1.9, *SD* = 0.3; mean arousal = 2.7, *SD* = 0.5). The 32 negative stimuli were equally divided between disgust-inducing (mean valence = 1.9, *SD* = 0.3; mean arousal = 2.6, *SD* = 0.4) and fear-inducing pictures (mean valence = 2.0, *SD* = 0.3; mean arousal = 2.7, *SD* = 0.5). Arousal did not differ significantly between negative and neutral pictures, *p* = .488. Within the negative set, fear- and disgust-inducing pictures did not differ significantly in either arousal, *p* = .447, or valence, *p* = .337.

#### 2.1.3. Procedure

Both experiments were programmed and administered using E-Prime 3.0 (Psychology Software Tools, 2018). Before beginning the task, participants were informed that they would be presented with neutral and negative emotional pictures and would perform a computational estimation task requiring them to approximate each sum without calculating its exact value. They were instructed to pay close attention to each picture before responding to the arithmetic problem. Participants were then introduced to the rounding-down and rounding-up strategies and asked to choose, for each problem, whichever of these two strategies produced the estimate closest to the exact sum. They were also informed that combining rounding down for one operand with rounding up for the other would generally yield the most accurate estimate (e.g., 20 + 50 for 21 + 47), but that this mixed-rounding strategy was not permitted.

Figure 1 provides an overview of the trial sequence. At the beginning of each trial, a fixation cross appeared for 1000 ms, followed by a neutral, disgust-inducing, or fear-inducing picture presented for 3000 ms. The picture was then replaced by an addition problem, which remained centered on the screen until the participant responded. To facilitate reliable identification of the strategy used on each trial, participants were required to state their answers aloud. After the participant responded, the experimenter clicked the mouse, which recorded response time and initiated two successive screens. On the first screen, the experimenter entered the participant’s answer. On the second, the strategy used was recorded. A blank white screen displayed for 3000 ms concluded each trial. Following a practice session, participants performed 64 experimental trials organized into two blocks of 32 trials each. The presentation order of the two blocks was counterbalanced across participants, and trials from the three emotion conditions were presented in random order within each block.

### 2.2. Results

Results are reported in two main parts. First, we examined how negative emotions influence strategy selection (i.e., better strategy selection, variability in strategy use, and determiners of strategy use). Second, we investigated the effects of negative emotions on participants’ performance (i.e., estimation latencies and percent deviations between estimates and exact results). Trials with latencies larger than the participant’s mean + 2.5 standard deviations were excluded (2.5%).

#### 2.2.1. Effects of Negative Emotions on Strategy Selection

*Better strategy selection*. Statistical analyses were conducted in R using the lme4 package and the glmer() function for generalized linear mixed-effects models. The effects of negative emotions on better strategy selection were analyzed at the trial level using a generalized linear mixed-effects model with a binomial distribution and logit link function. Better strategy selection was coded 1 when participants used the rounding-down strategy on rounding-down problems or the rounding-up strategy on rounding-up problems, and 0 otherwise. The model included Age (10–11 vs. 14–15 years), Emotion (neutral, disgust, fear), and their interaction as fixed effects, with participant and problem included as random intercepts (see Table 2 for means). The main effect of emotion was significant, *χ*^2^*(*2) = 22.55, *p* < .001. Holm-adjusted pairwise comparisons showed that participants selected the better strategy less often in the disgust condition than in the neutral condition (−6.0%; *β* = −0.338, *SE* = 0.091, *z* = −3.72, *p* < .001) and in the fear condition than in the neutral condition (−6.7%; *β* = −0.372, *SE* = 0.091, *z* = −4.11, *p* < .001). Better-strategy selection did not significantly differ between the disgust and fear conditions (*β* = −0.034, *SE* = 0.103, *z* = −0.33, *p* = .741). Neither the main effect of Age, *χ*^2^*(*1) = 0.60, *p* = .440, nor the Age × Emotion interaction, *χ*^2^(2) = 0.69, *p* = .707, was significant.

For exploratory purposes, we conducted an additional trial-level generalized linear mixed-effects analysis including Block (first vs. second block completed). The Block × Emotion interaction was significant, *χ*^2^(2) = 19.82, *p* < .001. Holm-adjusted pairwise comparisons showed that, in the second block, participants selected the better strategy less often in the disgust condition than in the neutral condition (−12.5%; *β* = −0.747, *SE* = 0.131, *z* = −5.71, *p* < .001) and in the fear condition than in the neutral condition (−10.3%; *β* = −0.630, *SE* = 0.132, *z* = −4.78, *p* < .001). In the first block, better-strategy selection did not significantly differ between the disgust and neutral conditions (*β* = −0.038, *SE* = 0.127, *z* = −0.30, *p* = .764) or between the fear and neutral conditions (*β* = −0.144, *SE* = 0.126, *z* = −1.15, *p* = .635).

*Variability in strategy use*. We asked whether participants solved addition problems using a single strategy or both available strategies, and whether this variability in strategy use changed under negative emotions. One way to address these issues is to tally the number of participants who used the harder rounding-up strategy (i.e., which requires incrementing the displayed decade digits before performing the estimation, unlike the easier rounding-down strategy that directly uses the displayed decade digits) on each problem in the neutral and negative emotion conditions. This enabled us to determine how many participants relied exclusively on one strategy, how many used the harder rounding-up strategy less frequently, and whether these patterns of use changed under negative emotions. Strategy variability is reported Table 3.

Table 3 describes variability in rounding-up strategy use. The participant-based analysis classifies participants according to the percentage of problems on which they used the rounding-up strategy. The problem-based analysis classifies problems according to the percentage of participants who used this strategy. In the overall sample, participant-based analyses showed a slight shift toward more frequent use of rounding-up in the negative condition: The percentage of participants using rounding-up on 67–100% of problems increased from 22% to 27%, whereas the percentage using it on 34–66% of problems decreased from 72% to 67%. Problem-based analyses similarly showed a shift toward greater use of rounding-up, with fewer problems for which only 0–33% of participants used rounding-up (28% vs. 9%) and more problems for which 34–66% of participants used it (25% vs. 41%) in the negative than in the neutral condition. This pattern was observed in both age groups and motivated the subsequent analyses directly examining strategy use as a function of emotion condition.

Use of the rounding-up strategy was analyzed at the trial level using a generalized linear mixed-effects model with a binomial distribution and logit link function. The model included Age (10–11 vs. 14–15 years), Emotion (neutral vs. negative), and their interaction as fixed effects, with participant and item/problem included as random intercepts (see Figure 2). The main effect of emotion was significant, *χ*^2^(1) = 14.98, *p* < .001. Participants used the rounding-up strategy more often in the negative condition than in the neutral condition (62.0% vs. 56.0%; *β* = 0.264, *SE* = 0.068, *z* = 3.87, *p* < .001). Descriptively, this increase was 7.7% in 10–11-year-old children and 4.2% in 14–15-year-old adolescents. However, the Age × Emotion interaction was not significant, *χ*^2^(1) = 1.56, *p* = .212.

*Determiners in strategy use*. Previous research on strategy use has shown that children calibrate the strategies they use to problem characteristics (for a review, see Siegler, 1996). The aims of the present analyses were to determine (a) whether negative emotions change the predictors of better strategy selection and, if so, (b) whether the influence of emotions on these predictors varies with age during childhood and the type of emotions. Problem-based correlations in each age group were calculated between the mean percentages of better strategy selection for each problem and three problem characteristics, in both the neutral and negative conditions. Problem characteristics included (a) the correct sum (e.g., 68 for 21 + 47), (b) the magnitude of the better estimate (e.g., 60 for 21 + 47), and (c) the difference between (1) the percentage of deviation between the estimate yielded by the rounding-down strategy and the exact sum and (2) the percentage of deviation between the estimate yielded by the rounding-up strategy and the exact sum (i.e., difference of deviations; e.g., 17.6 − 11.7 = 5.9% for 21 + 47). Correlations are presented in Table 4.

Correlations between problem characteristics and percent better strategy selection differed across the neutral and negative conditions. Mean percentages of better strategy selection correlated significantly with the difference of deviations (*r* = 0.536; *p* = .002) in the neutral condition, and with the magnitude of better estimate (*r* = 0.389, *p* = .028) in the negative condition. This pattern of results was observed in both age groups. 10–11- and 14–15-year olds’ better strategy selection correlated significantly with the difference of deviations in the neutral condition (*r* = 0.487, *p* = .005 and *r* = 0.492, *p* = .004, respectively), and with the magnitude of better estimate in the negative condition (*r* = 0.356, *p* = .046 and *r* = 0.393, *p* = .026, respectively).

These findings were further supported by stepwise regression analyses examining whether the problem characteristics that best predicted better strategy selection differed between the neutral and negative conditions, and across age groups. In the neutral condition, the difference of deviations emerged as the strongest and sole significant predictor, accounting for 28.7% of the variance (*R*^2^ = 0.287). The variables exact sum and magnitude of better estimate were excluded from the model because they did not explain additional variance beyond that accounted for by the difference of deviations. The same pattern was found in both age groups, with the difference of deviations being the only significant predictor of better strategy selection in both 10–11- and 14–15-year-old participants (*R*^2^ = 0.237 and *R*^2^ = 0.242, respectively). In the negative condition, the magnitude of better estimate significantly predicted the better strategy selection at the first step, accounting for 15.1% of the variance (*R*^2^ = 0.151), *F*(1, 30) = 5.34, *p* = .028. At the second step, the inclusion of the exact sum significantly improved the model, *ΔR*^2^ = 0.259, *ΔF*(1, 29) = 12.71, *p* = .001, increasing the total explained variance to 41.0% (*R*^2^ = 0.410). In the final model, both magnitude of better estimate (*β* = 1.222, *p* < .001) and exact sum (*β* = −.976, *p* = .001) were significant predictors. Although magnitude of better estimate and exact sum were strongly correlated (*r* = 0.854), collinearity diagnostics yielded tolerance values of 0.271 and VIF values of 3.69. Given the substantial correlation between these predictors, their unique contributions should nevertheless be interpreted with caution. The difference of deviations did not account for any additional unique variance and was therefore not retained in the final model. The same final model was retained for both age groups, with the magnitude of better estimate and exact sum explaining 32.5% of the variance in the 10–11-year-old participants and 43.8% of the variance in the 14–15-year-old participants.

#### 2.2.2. Effects of Negative Emotions on Performance

Estimation performance under the three emotion conditions was assessed using estimation latencies and percentage deviations between estimates and from the exact sums. Both measures were analyzed at the trial level using linear mixed-effects models. These analyses were conducted in R using the lme4 package and the lmer() function. The models included Age (10–11 vs. 14–15 years), Emotion (neutral, disgust, fear), and their interaction as fixed effects, with participant and item/problem included as random intercepts. Percentage deviation was computed using the formula [(exact sum − estimate)/exact sum] × 100. Thus, an estimate of 60 for the problem 21 + 47 corresponded to a deviation of [(68 − 60)/68] × 100 = 11.8% from the exact sum.

For estimation latencies, young children were significantly slower than older adolescents (6880 vs. 6011 ms, *F*(1, 62.38) = 5.30, *p* = .025.) Moreover, the main effect of emotion was significant, *F*(2, 2824.41) = 3.92, *p* = .020. Holm-adjusted pairwise comparisons showed that participants were significantly slower in the disgust than in the fear condition (+206 ms, *β* = 0.042, *SE* = 0.015, *t* = 2.75, *p* = .018). Estimation latencies did not significantly differ between the neutral and disgust conditions (*β* = −0.026, *SE* = 0.013, *t* = −2.03, *p* = .084) or between the neutral and fear conditions (*β* = −0.016, *SE* = 0.013, *t* = −1.19, *p* = .233). The Age × Emotion interaction was not significant, *F*(2, 2824.41) = 0.64, *p* = .528.

For percentage deviations, the main effect of emotion was significant, *F*(2, 4028) = 45.67, *p* < .001. Holm-adjusted pairwise comparisons showed larger deviations in the disgust condition than in the neutral condition (12.5% vs. 12.6%; *β* = 0.109, *SE* = 0.017, *z* = 6.54, *p* < .001) and in the fear condition than in the neutral condition (12.9% vs. 12.6%; *β* = 0.146, *SE* = 0.017, *z* = 8.75, *p* < .001). The difference between fear and disgust did not reach significance after Holm correction (*β* = 0.037, *SE* = 0.019, *z* = 1.92, *p* = .055). Neither the main effect of Age, *F*(1, 76.53) < 0.01, *p* > .999, nor the Age × Emotion interaction, *F*(2, 4028) < 0.01, *p* > .999, was significant.

In conclusion, we found that negative emotions led participants to select the better strategy less often, particularly during the second block of the experiment, and the rounding-up strategy more frequently. Importantly, problem characteristics associated with better strategy selection differed between the neutral and negative emotion conditions. Participants relied on the difference of deviations to select the problem-solving strategy in neutral condition, whereas they relied on the magnitude of the better estimate, and therefore on the size of the problem, in negative conditions. Better-strategy selection did not significantly differ between fear and disgust conditions, and no significant Age × Emotion interaction was observed. We also found that negative emotions led participants to provide poorer estimates of addition problems. No effects of negative emotions were observed on estimation latencies. It should be noted, however, that in Experiment 1, strategy execution was not assessed independently of strategy selection. To compare participants’ performance under negative and neutral emotion conditions, and to determine if the effects of emotions on participants’ performance vary with the type of negative emotions, the type of strategy and participants’ age, performance should be assessed under negative and neutral conditions for each strategy independently of strategy selection. This was the goal of Experiment 2.

## 3. Experiment 2: Effects of Negative Emotions on Strategy Execution in Children and Adolescents

### 3.1. Method

Experiment 2 used the same arithmetic problems, emotional pictures, and trial sequence as Experiment 1 (see Figure 1). Unlike in Experiment 1, however, participants did not choose between strategies but were instructed which rounding strategy to apply. The 64 addition problems were distributed across two matched subsets, each containing an equal number of problems for which rounding down or rounding up yielded the better estimate. Children aged 10–11 years applied the rounding-down strategy to all problems, whereas adolescents aged 14–15 years applied the rounding-up strategy. For both age groups, each subset was completed once in the neutral condition and once in the negative emotion condition, with presentation order randomized. This choice was made to control for baseline performance differences between 10–11- and 14–15-year-old participants, as the rounding-down strategy is easier to execute than the rounding-up strategy. Such a control aimed at maximally matching task difficulty across age groups, as age differences in effects of emotions may be contaminated by relative difficulty of the task. However, this design necessarily confounded age with the strategy executed, a limitation that should be considered when interpreting age-related effects.

At the end of each trial, participants were asked to rate the valence of the picture they had just seen using a Likert scale ranging from 1 (very negative) to 9 (very positive). This procedure was implemented because, although the disgust- and fear-inducing pictures were matched on valence based on the normative ratings provided by the NAPS, we aimed to verify that these two negative emotion categories did not significantly differ in perceived valence. This step was critical to ensure that any observed differences between negative emotional conditions could not be attributed to differences in valence. The valence ratings revealed that disgust pictures were evaluated by our participants as more negative than fear pictures (2.7 vs. 3.5, respectively, *p* = .002). To compare fear and disgust with overall matched valences, we excluded six trials in our analyses of participants’ performance. These six trials corresponded to the fear pictures that received the least negative valence ratings. Following these exclusions, valence ratings did not significantly differ between the fear and disgust conditions (2.7 vs. 3.0, respectively, *p* = .122). (2.7 vs. 3.0, respectively, *p* = .122).^2^

### 3.2. Results

Mean estimation latencies and response accuracy were analyzed at the trial level using mixed-effects models. Estimation latencies were analyzed using a linear mixed-effects model, whereas response accuracy was analyzed using a generalized linear mixed-effects model with a binomial distribution and logit link function. These analyses were conducted in R using the lme4 package, with the lmer() function for estimation latencies and the glmer() function for response accuracy. Both models included Age (10–11 vs. 14–15 years), Emotion (neutral, disgust, fear), and their interaction as fixed effects, with participant included as random intercepts (see Table 5 for means). For each trial, response was coded as correct (1) when the participant’s estimate matched the expected result obtained with the instructed strategy and as incorrect (0) otherwise. Trials with response times exceeding 2.5 standard deviations above each participant’s mean were excluded from the analyses (1.5% of trials).

For estimation latencies, the main effect of emotion was significant, *F*(2, 4431.12) = 101.11, *p* < .001. Participants were slower in the disgust condition than in the neutral condition (+386 ms; *β* = 0.096, *SE* = 0.007, *z* = 13.24, *p* < .001) and in the fear condition than in the neutral condition (+330 ms; *β* = 0.075, *SE* = 0.009, *z* = 8.69, *p* < .001). Participants were also significantly slower in the disgust than in the fear condition (+56 ms, *β* = 0.022, *SE* = 0.010, *z* = 2.24, *p* = .025). Neither the main effect of age, *F*(1, 78.90) = 0.29, *p* = .591, nor the Age × Emotion interaction, *F*(2, 4431.12) = 2.55, *p* = .078, were significant.

Given the significant Block × Emotion interaction observed in Experiment 1, revealing larger effects of negative emotions in the second than in the first block on strategy selection, we conducted an additional trial-level linear mixed-effects analysis of estimation latencies in Experiment 2. The aim was to determine whether the block-related differences in emotion effects observed for strategy selection in Experiment 1 would also emerge for strategy execution. In contrast to Experiment 1, the Block × Emotion interaction was not significant, *F*(2, 4430.09) = 1.99, *p* = .137, providing no evidence that the effects of negative emotions on estimation latencies differed between the two blocks.

For response accuracy, the main effect of emotion was not significant, *χ*^2^(2) = 1.78, *p* = .411, whereas the main effect of age, *χ*^2^(1) = 3.94, *p* = .047, and the Age × Emotion interaction, *χ*^2^(2) = 9.18, *p* = .010, were significant. Holm-adjusted pairwise comparisons conducted within each age group showed that 10–11-year-old children were less accurate in the disgust condition than in the neutral condition (−1.5%, *β* = −0.886, *SE* = 0.329, *z* = −2.70, *p* = .042). Accuracy did not significantly differ between the fear and neutral conditions or between the disgust and fear conditions in this age group (both *ps* ≥ .412). Among 14–15-year-old adolescents, none of the pairwise comparisons between emotion conditions were significant (all *ps* ≥ .920).

To summarize, Experiment 2 showed that both disgust and fear slowed the execution of instructed strategies relative to the emotionally neutral condition, with disgust producing a greater deleterious effects than fear. Negative emotions also affected response accuracy differently across age groups, with disgust reducing accuracy relative to the neutral condition in 10–11-year-old children but not in 14–15-year-old adolescents.

## 4. General Discussion

The present study aimed to determine (a) the mechanisms by which negative emotions disrupt the strategic aspects of children’s and adolescents’ arithmetic performance, (b) whether different types of negative emotions differentially influence these strategic aspects, and (c) how these effects of negative emotions change with children’s age. Children aged 10–11 years and adolescents aged 14–15 years estimated the sums of two-digit addition problems presented after neutral, disgust-inducing, or fear-inducing pictures. They either chose between rounding down and rounding up to obtain the better estimate (Experiment 1) or applied an instructed rounding strategy (Experiment 2). Negative emotions disrupted arithmetic performance by decreasing better-strategy selection, altering the pattern of problem characteristics associated with strategy choices, increasing the use of the rounding-up strategy, and impairing strategy execution. Although fear and disgust did not significantly differ in their effects on strategy selection, emotion-specific effects emerged for strategy execution, with disgust producing greater slowing than fear. Age did not significantly modulate the effects of emotion on better-strategy selection or strategy execution latencies, although age-related differences emerged for response accuracy. Together, these findings provide new evidence that negative emotions affect multiple components of strategic aspects of arithmetic performance, and that some of these effects vary according to the specific emotion and performance measure.

### 4.1. How Negative Emotions Influence Better Strategy Selection in Children and Adolescents?

Lemaire (2026) found that negative emotions led children to select the better strategy less often on each problem. Unknown was whether this decrement in better strategy selection under negative emotions hold for different negative emotions and whether this stems from children being less systematic in strategy choices and/or basing their strategy selection on different problem characteristics. Experiment 1 showed that both sources contribute to deleterious effects of negative emotions on children’s better strategy selection.

We found that fear and disgust led children and adolescents to select the better strategy less often to estimate the sums of two-digit addition problems. These findings replicate the deleterious effects of negative emotions on strategy selection previously observed in arithmetic in adults (Lallement & Lemaire, 2025; Lemaire, 2024), and more recently in children (Lemaire, 2026). Moreover, the deleterious effects of emotions were larger in Block 2 (the last 32 arithmetic problems) than in Block 1 (the first 32 arithmetic problems). One possible explanation for this pattern is that, if negative emotions influence participants by capturing their attentional and cognitive resources (Okon-Singer et al., 2015; Pessoa, 2009; Verbruggen & De Houwer, 2007), their effects could be amplified toward the end of the experiment, when participants may experience experimental fatigue or resource depletion. Interestingly, this interaction between the block and emotion was only found on the ability to select the better strategy for solving arithmetic problems (Expt. 1), but not on strategy execution (Expt. 2). This pattern could indicate that, if fatigue or resource depletion occurred, it may have exacerbated the influence of negative emotions on processes specifically involved in selecting the better strategy, rather than on executing strategy components once a strategy is chosen on a given item. When participants have to find the better estimates as in Experiment 1, they have to select and execute the better strategy. Selecting the better strategy requires analyzing problem features (e.g., size of unit digits) and calculating relative costs/benefits of available strategies. Thus, one possibility is that strategy selection mechanisms are particularly susceptible to the effects of negative emotions as cognitive resources become increasingly taxed over the course of the experiment. However, as fatigue was not directly assessed in the present study, this interpretation remains speculative.

One of the key findings of this study was that different patterns of associations between problem features and better strategy selection were observed in the neutral and negative emotion conditions. In the emotionally neutral condition, participants relied on the difference between the percentage deviation of the rounding-down estimate from the exact sum and the percentage deviation of the rounding-up estimate from the exact sum (i.e., difference of deviations). The difference of deviations constitutes an optimal problem feature as it captures the relative accuracy of the two rounding strategies by quantifying which strategy yields an estimate closer to the exact sum. As such, it provides the most diagnostic information for better strategy selection. This pattern suggests that, under neutral emotions, participants were able to rely on this diagnostic information when selecting between strategies. In contrast, under negative emotions, better-strategy selection was no longer significantly associated with the difference of deviations but was instead predicted by the magnitude of the better estimate and the exact sum. These variables were not relevant for strategy selection, as problems best solved with the rounding-down strategy and those best solved with the rounding-up strategy were matched on these magnitude-related problem features. Unlike the difference of deviations, which directly reflects the relative accuracy of the two strategies, the magnitude of the better estimate and the sum are salient numerical characteristics, directly related to the magnitude of operands, that are immediately available during problem encoding. Although this information may appear relevant because larger problems are often perceived as more difficult, it does not indicate which rounding strategy will produce the more accurate estimate. Thus, the observed pattern suggests that negative emotions may shift strategy selection away from diagnostic information toward salient but nondiagnostic magnitude-related cues, which may contribute to the observed decrease in better-strategy selection.

These results are consistent with, but do not directly demonstrate, our first hypothesis that negative emotions may reduce reliance on relevant problem characteristics and promote reliance on more salient but less informative features, such as problem size. This shift under negative emotions could explain the disruption in participants’ ability to select the better strategy to solve arithmetic problems. Importantly, it also helps explain why participants chose the rounding-up strategy more frequently under negative emotions. When participants no longer base their strategy choices on the relative accuracy of the two strategies but instead rely on the more salient characteristic of problem size, the rounding-up strategy appears to become the default strategy, particularly for larger problems. Thus, the increased use of the rounding-up strategy likely reflects a change in the information guiding strategy selection rather than a deliberate preference for this more demanding strategy. Interestingly, this increase in rounding-up choices cannot be explained by a tendency to favor the easier strategy under negative emotions, as the rounding-up strategy is computationally more demanding than the rounding-down strategy (e.g., Lemaire, 2024; Lemaire et al., 2004). Instead, it is more consistent with the idea that negative emotions alter the information on which strategy choices are based.

### 4.2. Effects of Discrete Negative Emotions on Strategic Aspects of Children’s Arithmetic Performance and Age-Related Differences Therein

Another goal of the present study was to determine whether distinct negative emotions differentially influence strategic aspects of children’s arithmetic performance, and whether these effects change with age.

Our findings replicate the deleterious effects of negative emotions on the ability to select the better strategy to solve arithmetic problems (Lallement & Lemaire, 2025; Lemaire, 2024, 2026), as well as their broader detrimental influence on arithmetic performance in adults (Framorando & Gendolla, 2018, 2019; Kleinsorge, 2007, 2009; Lallement & Lemaire, 2021, 2025; Lemaire, 2024; Liu et al., 2021; Schimmack & Derryberry, 2005; Zhu et al., 2021, 2022, 2024), and in children (Kucian et al., 2018; Lemaire, 2022, 2026; Rubinsten & Tannock, 2010; Viesel-Nordmeyer & Lemaire, 2025). They also provide additional evidence that the effects of emotions persist even when all strategic aspects of performance, such as strategy repertoire and selection, are experimentally controlled (Lallement & Lemaire, 2025; Lemaire, 2024, 2026). Importantly, the present findings revealed different patterns for strategy selection and strategy execution. In Experiment 1, both disgust and fear led children and adolescents to select the better strategy less frequently than in the neutral condition, with no significant difference between the two negative emotions. In Experiment 2, children and adolescents were slower to execute the instructed strategies under both disgust and fear than under neutral emotions. Moreover, they were significantly slower under disgust than under fear. Thus, our second hypothesis that the effects of negative emotions on strategic aspects of arithmetic performance would differ as a function of discrete emotions was supported for strategy execution but did not hold for strategy selection.

The larger slowing under disgust than fear provides evidence that discrete negative emotions can differentially affect some strategic aspects of arithmetic performance in children and adolescents. Interestingly, this pattern in children and adolescents differs from that previously observed in adults. Lallement and Lemaire (2025) found that disgust, but not fear, impaired better-strategy selection, whereas both emotions slowed strategy execution, with fear producing a greater slowing effect than disgust. In the present study, disgust and fear did not significantly differ in their effects on better-strategy selection, whereas disgust produced greater slowing than fear during strategy execution. Taken together, these findings suggest that emotion-specific effects may depend on the strategic component considered and may differ across development. One possible explanation for the different patterns observed in the present study and in adults is that the effects of disgust and fear on arithmetic performance may become more differentiated with development. Although both emotions are associated with avoidance of aversive or threatening situations (Cisler et al., 2009, for a review), their cognitive and functional consequences may become more specialized with development. As executive functions, emotion regulation, and emotion differentiation continue to mature throughout adolescence, children and adolescents may process disgust- and fear-inducing stimuli in a more similar manner, potentially resulting in less differentiated effects on arithmetic strategy selection and execution. By contrast, adults may recruit more differentiated cognitive and regulatory processes when experiencing these emotions, resulting in the distinct patterns observed by Lallement and Lemaire (2025). Interestingly, the difference between the present findings in children and those reported in adults raises the possibility that the effects of discrete negative emotions undergo developmental changes. Rather than reflecting stable emotion-specific mechanisms across the lifespan, the influence of disgust and fear on strategic aspects of performance may change with age. However, this interpretation should be considered with caution, as our experimental design did not establish whether the pictures specifically elicited the intended discrete emotions. Although the differential effects of disgust and fear on strategy execution suggest that the two emotion conditions did not have identical effects on performance, direct measures of the emotions elicited by the pictures would be necessary to determine whether these differences specifically resulted from the intended discrete emotions. Moreover, although valence ratings did not significantly differ between the disgust and fear conditions, we cannot entirely rule out the possibility that residual differences in valence contributed to the observed difference in strategy execution.

To rule out the possibility that the observed effects were merely driven by differences in arousal (i.e., intensity) rather than by valence, we conducted additional analyses that are reported in the Supplementary Materials. Specifically, negative trials were divided into low- and high-arousal conditions, collapsing over disgust and fear. These analyses revealed no reliable differences between low- and high-arousal negative emotion conditions, both for strategy selection in Experiment 1 and strategy execution in Experiment 2. These results suggest that the reported effects of negative emotions are not driven by arousal but are primarily related to emotional negative valence.

Finally, negative emotions led both age groups to select the better strategy less frequently and to slow down when estimating addition problems, with no significant Age × Emotion interaction for better strategy selection or strategy execution latencies. However, age-related differences emerged for response accuracy in Experiment 2. Specifically, disgust reduced response accuracy relative to the neutral condition in 10–11-year-old children but not in 14–15-year-old adolescents. Thus, our third hypothesis of age-related differences in the effects of negative emotions on children’s and adolescents’ arithmetic performance was partially supported, with age-related differences emerging for response accuracy but not for better-strategy selection or strategy execution latencies. These findings partially converge with recent evidence showing developmental differences in the effects of negative emotions on arithmetic performance (Lemaire, 2022, 2026; Viesel-Nordmeyer & Lemaire, 2025). However, these findings should be interpreted with caution because age was confounded with the strategy executed in Experiment 2, with younger children executing the rounding-down strategy and adolescents executing the rounding-up strategy. Previous studies reported that the disruptive effects of emotions on strategy selection and execution decrease with age, a pattern attributed to greater arithmetic fluency as well as improvements in executive and emotional regulation mechanisms with age. Interestingly, the present study provided only partial evidence for such an age-related decrease between 10 and 15 years of age. Whereas the effect of disgust on response accuracy was observed in younger children but not in adolescents, the effects of negative emotions on better-strategy selection and strategy execution latencies did not significantly differ between age groups. This pattern does not necessarily imply that emotional influences on strategic processing are already mature by late childhood. Rather, one possibility is that developmental gains in executive functions, metacognitive abilities, and emotion regulation may reduce some effects of negative emotions without being sufficient to counteract others. Importantly, this account assumes that adolescents engage these regulation mechanisms when performing the task. In the present study, although adolescents outperformed younger children on some aspects of performance, age did not significantly modulate the effects of disgust and fear on better-strategy selection or strategy execution latencies. One possible explanation is that, because the arithmetic task was relatively easy for adolescents, they may not have perceived a sufficient need to recruit emotional regulation strategies, which could account for the absence of age-related differences on these measures. An alternative explanation concerns the specific set of negative emotions examined in the present study. Previous studies typically relied on a broader range of negative emotions, including for example sadness or anger, which may be more sensitive to developmental differences in effects of emotions and emotional regulation. In contrast, disgust and fear, as highly salient emotions promoting avoidance of aversive situations, are more strongly associated with automatic attentional capture and may therefore be less modulated by age-related improvements in emotion regulation processes, potentially contributing to the absence of significant age-related differences in better strategy selection and strategy execution latencies.

The present study has several limitations. First, future studies should extend the range of emotions examined beyond disgust and fear to include other negative emotions such as anxiety, anger, or sadness, as well as positive emotions such as joy. Relying on a richer set of emotions may reveal different effects of emotions on arithmetic performance and allow for a more direct test of theoretical accounts, such as the prediction that activating and deactivating emotions exert opposite influences on performance. Importantly, such an approach may also help uncover age-related differences that are emotion-specific. Second, future studies should directly assess the efficiency of emotional regulation mechanisms in comparable arithmetic tasks. Doing so would make it possible to determine whether the deliberate engagement of emotional regulation processes can decrease the deleterious effects of emotions observed in the present study on both strategy selection and execution, and to more directly test the hypothesis that developmental differences in effects of emotions stem from age-related improvements in emotion regulation efficiency. This could be achieved by instructing children to regulate their emotions during task performance, for instance by encouraging cognitive reappraisal of emotional pictures or by guiding attentional deployment away from emotionally salient regions of the pictures. Third, the validity of the emotional manipulation could be more directly assessed in future studies. Previous research has used emotion judgment or recognition tasks to verify that participants processed the emotional stimuli as intended (Zhu et al., 2021; Zhu et al., 2022; Lallement & Lemaire, 2021; Lemaire, 2024). In the present study, no such behavioral measure was included. Future studies could therefore incorporate an emotion judgment or recognition task to provide a more direct verification of the effectiveness of the emotional manipulation. In addition, valence ratings were collected only in Experiment 2, preventing us from directly verifying whether the fear and disgust stimuli were matched on valence in Experiment 1. Moreover, although participants rated the valence of the pictures, these ratings did not establish whether the pictures specifically elicited the intended discrete emotions (i.e., fear or disgust). Future studies should therefore also assess the specific emotion elicited by each stimulus to more directly validate the discrete-emotion manipulation. Fourth, the absence of significant differences between fear and disgust for some measures, as well as the absence of significant Age × Emotion interactions for better-strategy selection and strategy execution latencies, should be interpreted cautiously, as the present study may not have been sensitive to smaller effects. Thus, nonsignificant findings should be interpreted as an absence of evidence for differences rather than as evidence that the effects of emotion were equivalent across emotion types or age groups.

## 5. Conclusions

In this study, negative emotions impaired children’s and adolescents’ arithmetic performance by disrupting strategic aspects of problem solving. More specifically, fear and disgust led both 10–11-year-old and 14–15-year-old participants to select the better strategy less frequently and to execute strategies less efficiently. Importantly, the pattern of problem characteristics associated with better-strategy selection differed across emotion conditions: Selection was associated with the diagnostic difference-of-deviations cue in the neutral condition, whereas magnitude-related, nondiagnostic problem characteristics were predictive under negative emotions. This pattern suggests that negative emotions may shift strategy selection away from diagnostic information toward salient but nondiagnostic problem information. Moreover, the effects of negative emotions varied depending on the strategic aspect considered. Fear and disgust did not significantly differ in their effects on better-strategy selection, whereas children and adolescents were slower to execute instructed strategies under disgust than under fear. Age did not significantly modulate the effects of negative emotions on better-strategy selection or strategy execution latencies, but age-related differences emerged for response accuracy, with disgust reducing accuracy relative to the neutral condition in 10–11-year-old children but not in 14–15-year-old adolescents. However, this age-related finding should be interpreted cautiously given the age-strategy confound in Experiment 2. These findings extend previous research by identifying specific mechanisms through which emotions influence children’s and adolescents’ arithmetic performance. They further suggest that the strategy approach adopted here, combining assessments of strategy selection with controlled examinations of strategy execution, provides a relevant approach for furthering our understanding of how effects of emotions on cognitive performance change across different developmental stages and cognitive domains. These findings may also have implications for mathematics education by highlighting the importance of considering children’s and adolescents’ emotional states during mathematical activities. Raising teachers’ awareness of these effects and helping students develop effective emotion regulation strategies may contribute to limiting the disruptive effects of negative emotions on learning, although these possibilities need to be directly examined in educational settings.

## Figures and Tables

**Figure 1:**
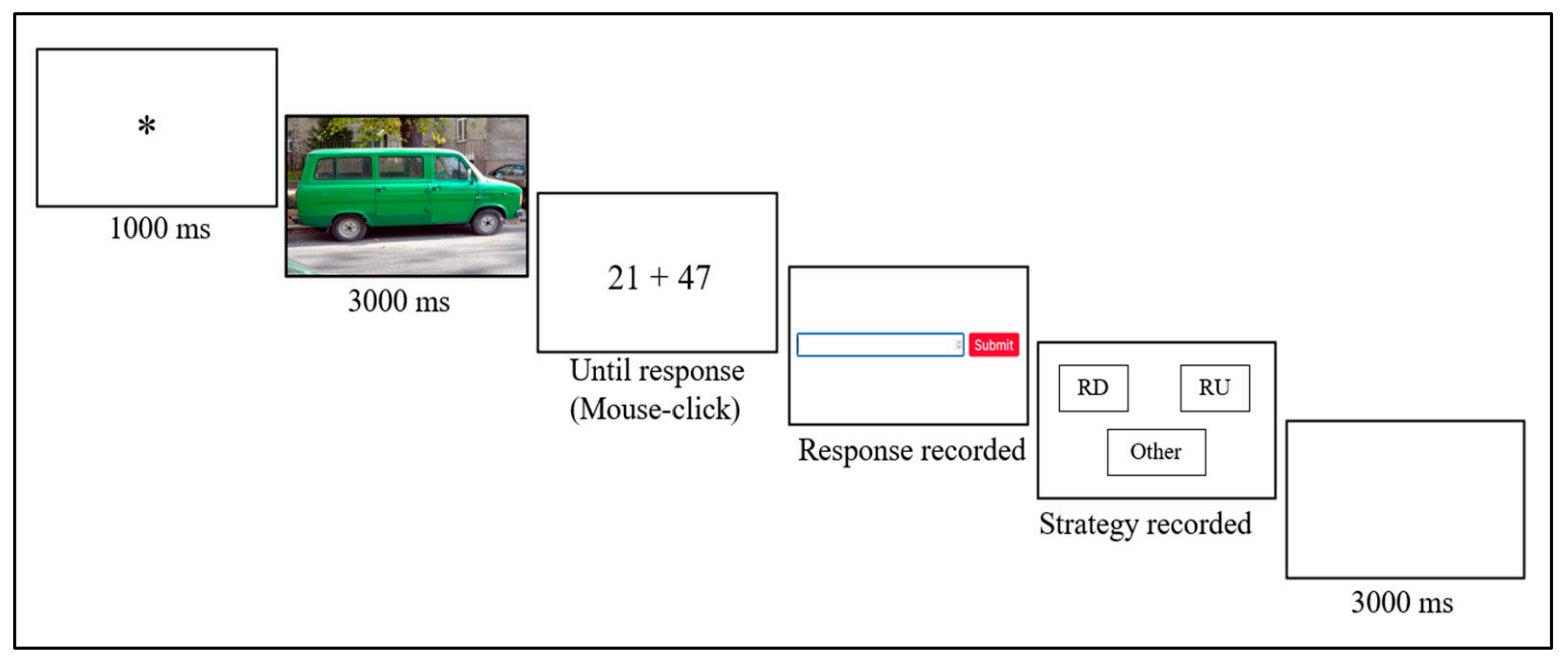
Sequence of events within an example of emotionally neutral trial.

**Figure 2:**
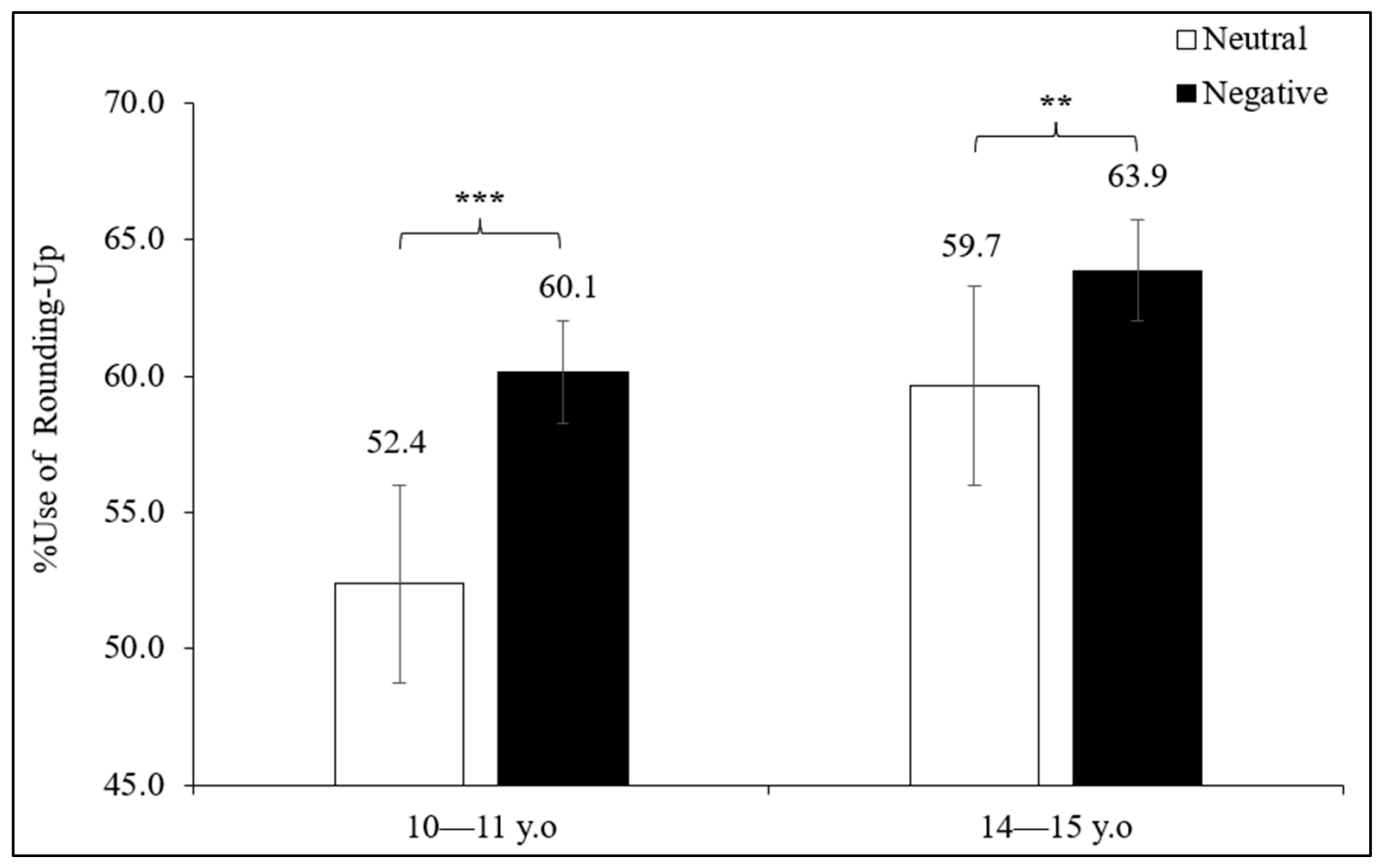
Mean percentages of use of the rounding-up strategy as a function of age (10–11 y.o, 14–15 y.o) and emotional condition (neutral, negative). ** *p* < .01; *** *p* < .001.

**Table 1:** Participants’ characteristics.

Characteristics	10–11 y.o	14–15 y.o	*Fs*
Experiment 1: Strategy selection			
*N* (female/male)	32 (15/17)	32 (18/14)	-
Mean age in y.o. (*SD*)	10.9 (0.4)	14.9 (0.3)	-
Arithmetic fluency (*SD*)	85.2 (21.3)	103.2 (18.6)	13.008 ^c^
Experiment 2: Strategy execution			
*N* (female/male)	32 (18/14)	32 (21/11)	-
Mean age in y.o. (*SD*)	10.8 (0.3)	14.9 (0.3)	-
Arithmetic fluency (*SD*)	91.5 (11.8)	124.2 (16.6)	82.415 ^c^

Note. ^c^ *p* < .001. Arithmetic fluency was assessed using a paper-and-pencil arithmetic test (Tempo Test Rekenen; De Vos, 1992). The test consists of four sets of 40 arithmetic problems: one set of additions, one of subtractions, one of multiplications, and one mixed set including all three operations. For each set, participants had to provide the correct answer to as many problems as possible in 1 min. The sum of correctly solved problems was calculated for each individual.

**Table 2:** Mean percentages of better strategy selection as a function of age (10–11 y.o, 14–15 y.o.) and emotion (neutral, disgust, fear) in Experiment 1.

	Neutral	Disgust	Fear	Disgust-Neutral	Fear-Neutral	Disgust-Fear
Overall	75.2	69.2	68.5	−6.0 ^c^	−6.7 ^c^	0.7
10–11 y.o	77.1	72.0	69.7	−5.1 ^a^	−7.4 ^b^	2.3
14–15 y.o	73.2	66.5	67.3	−6.7 ^b^	−6.0 ^b^	−0.8

Note. ^a^ *p* < .05; ^b^ *p* < .01; ^c^ *p* < .001.

**Table 3:** Distribution of use of the rounding-up strategy across participants and problems, in the neutral condition and in the negative condition, in Experiment 1.

	Neutral Condition			Negative Condition		
	0–33%	34–66%	67–100%	0–33%	34–66%	67–100%
	Participant-based analyses					
Overall	6	72	22	6	67	27
10–11 y.o	6	81	13	6	75	19
14–15 y.o	6	63	31	6	59	34
	Problem-based analyses					
Overall	28	25	47	9	41	50
10–11 y.o	38	16	47	19	38	44
14–15 y.o	22	31	47	3	47	50

Note. Percentages may not sum to exactly 100% because of rounding. Each entry refers to the percentages of individuals who used the rounding-up strategy on between 0–33%, 34–66%, and 67–100% of problems and the percentages of problems on which the rounding-up strategy was used by 0–33%, 34–66%, and 67–100% of individuals; For examples, 6% of participants used the rounding-up strategy on between 0–33% of problems, and 28% of problems were solved with the rounding-up strategy by between 0–33% of individuals.

**Table 4:** Correlations between mean percentages of better strategy selection and problem characteristics (exact sum, magnitude of better estimate and difference of deviations) overall and in each age group.

Variable	Neutral Condition				Negative Condition			
	1	2	3	4	1	2	3	4
	Overall							
1. Better strategy selection		−0.120	0.264	0.536 ^b^		0.067	0.389 ^a^	0.291
2. Exact sum			0.819 ^c^	−0.159			0.854 ^c^	−0.252
3. Better estimate				0.039				−0.018
4. Difference of deviations								
	10–11 y.o							
1. Better strategy selection		−0.033	0.199	0.487 ^b^		0.072	0.356 ^a^	0.340
2. Exact sum			0.819 ^c^	−0.159			0.854 ^c^	−0.252
3. Better estimate				0.039				0.164
4. Difference of deviations								
	14–15 y.o							
1. Better strategy selection		−0.181	0.281	0.492 ^b^		0.059	0.393 ^a^	0.231
2. Exact sum			0.819 ^c^	−0.159			0.854 ^c^	−0.252
3. Better estimate				0.039				−0.018
4. Difference of deviations								

Note. ^a^ *p* < .05; ^b^ *p* < .01; ^c^ *p* < .001.

**Table 5:** Estimation latencies (ms) and percentages of correct responses as a function of Age (10–11 y.o, 14–15 y.o), and Emotion (neutral, disgust, fear), in Experiment 2.

	Neutral	Disgust	Fear	Disgust-Neutral	Fear-Neutral	Disgust-Fear
	Estimation latencies (ms)					
Overall	4335	4721	4665	386 ^a^	330 ^a^	56
10–11 y.o	4621	4927	4920	306 ^a^	299 ^a^	7
14–15 y.o	4049	4516	4410	467 ^a^	361 ^a^	106
	Percentages of correct responses					
Overall	98.1	97.6	98.3	−0.5	0.2	−0.7
10–11 y.o	98.7	97.2	99.1	−1.5	0.4	−1.9
14–15 y.o	97.5	98.0	97.5	0.5	0.0	0.5

Note.; ^a^ *p* < .001.

## Data Availability

Data can be found on the following link: https://osf.io/b5jah/overview?view_only=a95ff3abca9e42f3b3a35a8d9397468f (assessed on 5 October 2026).

## Notes

1 Because the a priori power analysis was based on the overall effect of emotion rather than on the specific emotion-type and age-related contrasts, sensitivity analyses were also conducted based on the final sample size. A sensitivity analysis conducted in G*Power indicated that, with *N* = 64, *α* = 0.05, and 80% power, the minimum detectable effect for the within-participant fear–disgust contrast was Cohen’s *dz* = 0.36. For the Age × Emotion interaction, the minimum detectable effect was Cohen’s *f* = 0.16 (*η*^2^*p* ≈ 0.025). Thus, the study may not have been sufficiently sensitive to detect effects smaller than these thresholds.

2 Analyses conducted on the full set of trials, including the six fear trials excluded to match the fear and disgust conditions on valence, yielded the same pattern of effects as the analyses reported here. We report the analyses based on the valence-matched subsets to minimize the possibility that differences in valence between fear and disgust could confound comparisons between the two negative emotion conditions.

## Supplementary Materials

The following supporting information can be downloaded at https://www.mdpi.com/article/10.3390/jintelligence14100255/s1. Table S1. Statistics of effects on *mean* percentages of better strategy selection. Table S2. Mean percentages of better strategy selection as a function of Age (10–11 y.o, 14–15 y.o) and Emotion (neutral, high-arousal negative, low-arousal negative). Table S3. Statistics of effects on estimation latencies and percentages of correct responses. Table S4. Estimation latencies (ms) and percentages of correct responses as a function of Age (10–11 y.o, 14–15 y.o), and Emotion (neutral, high-arousal negative, low-arousal negative).
