# Supplementary material for: How Disgust and Fear Influence Strategic Aspects of Arithmetic Performance in Children and Adolescents
Source: J Intell. 2026 Oct 11;14(10):255. doi: 10.3390/jintelligence14100255 (PMC13706484; doi:10.3390/jintelligence14100255)
Supplement: Supplementary file 1 [file jintelligence-14-00255-s001.zip › jintelligence-4497943 supplementary.pdf]

Supplementary material: Additional analyses examining the effects of arousal on strategy selection and strategy execution.

To rule out the possibility that the observed effects of emotions were merely driven by differences in arousal (i.e., intensity) rather than valence, we conducted additional ANOVAs involving 2 (Age: 10—11 y.o, 14—15 y.o) × 3 (Emotion: neutral, high-arousal negative, low-arousal negative) on percentages of better strategy selection in Experiment 1 and on estimation latencies and percentages of correct responses in Experiment 2.

Experiment 1: Strategy selection

Table S1. Statistics of effects on *mean* percentages of better strategy selection.

Effects	*MSe*	*F*	*p*	η^2p
Age	308.371	.818	.369	.013
Emotion	57.263	20.485	<.001	.248
Age × Emotion	57.263	.106	.863	.002

Table S2. Mean percentages of better strategy selection as a function of Age (10—11 y.o, 14—15 y.o) and Emotion (neutral, high-arousal negative, low-arousal negative).

	Neutral	High arousal	Low arousal	High arousal–Neutral	Low arousal–Neutral	High arousal – Low arousal
Overall	75.2	70.4	67.4	-4.8[c]	-7.8[c]	3.0
10—11 y.o	77.1	72.1	69.7	-5.0[c]	-7.4[c]	2.4
14—15 y.o	73.2	68.6	65.2	-4.6[b]	-8.0[c]	3.4

Note. [a] $p < .05$; [b] $p < .01$; [c] $p < .001$.

The main effect of emotions on better strategy selection was significant. Participants selected the better strategy less frequently in both the high-arousal negative condition (-4.8 %), $p < .001$, and the low-arousal negative condition (-7.8 %), $p < .001$, than in the neutral condition. In contrast, the difference between the high-arousal negative and the low-arousal negative conditions did not reach conventional levels of statistical significance, $p = .055$, suggesting that the deleterious effects of negative emotions on strategy selection were primarily driven by emotional valence rather than arousal. Although the *p*-value of .055 could be considered marginal, the descriptive pattern indicates a larger decrease in better strategy selection in the low-arousal negative condition than in the high-arousal negative condition, which runs counter to what would be expected if the observed effects were primarily driven by arousal.

Experiment 2: Strategy execution

Table S3. Statistics of effects on estimation latencies and percentages of correct responses.

Effects	*MSe*	*F*	*p*	η^2p
	Estimation latencies (ms)			
Age	789567.356	4.830	.032	.072
Emotion	62499.014	45.209	<.001	.422
Age × Emotion	62499.014	1.392	.253	.022
	Percentages of correct responses			
Age	5.956	.638	.427	.010
Emotion	14.096	.125	.883	.002
Age × Emotion	14.096	3.325	.039	.051

Table S4. Estimation latencies (ms) and percentages of correct responses as a function of Age (10—11 y.o, 14—15 y.o), and Emotion (neutral, high-arousal negative, low-arousal negative).

	Neutral	High arousal	Low arousal	High arousal – Neutral	Low arousal – Neutral	High arousal – Low arousal
				Estimation latencies (ms)		
Overall	4335	4690	4707	355[c]	372[c]	-17
10—11 y.o	4621	4920	4924	299[c]	303[c]	-4
14—15 y.o	4049	4461	4490	412[c]	441[c]	-29
				Percentages of correct responses		
Overall	98.1	97.9	97.8	-0.2	-0.3	0.1
10—11 y.o	98.7	98.8	97.0	0.1	-1.7	1.8
14—15 y.o	97.5	97.1	98.5	-0.4	1.0	-1.4

Note. [a] $p < .05$; [b] $p < .01$; [c] $p < .001$.

The main effect of emotion was significant on estimation latencies. Participants were slower in both the high-arousal negative condition (+355 ms; $p < .001$), and the low-arousal negative condition (+372 ms; $p < .001$), compared to the neutral condition However, the slowing did not significantly differ between the high-arousal and low-arousal negative conditions, $p = .698$, suggesting that the deleterious effects of negative emotions observed on estimation latencies were primarily driven by emotional valence rather than arousal.
